# Group versus individual treatment for substance use disorders: a study protocol for the COMDAT trial

**DOI:** 10.1186/s12889-021-10271-4

**Published:** 2021-02-26

**Authors:** Sidsel Helena Karsberg, Mads Uffe Pedersen, Morten Hesse, Birgitte Thylstrup, Michael Mulbjerg Pedersen

**Affiliations:** Centre for Alcohol and Drug Research, Bartholins Allé 10, 8000 Aarhus C, Denmark

**Keywords:** Substance use disorders, Group-based treatment, Individual treatment, Randomized controlled trial, Community based treatment, Contingency management

## Abstract

**Background:**

Alcohol and other drug use disorders contribute substantially to the global burden of illness. The majority of people with substance use disorders do not receive any treatment for their problems, and developing treatments that are attractive and effective to patients should be a priority. However, whether treatment is best delivered in a group format or an individual format has only been studied to a very limited degree. The COMDAT (Combined Drug and Alcohol Treatment) trial evaluates the feasibility, acceptability, and cost effectiveness of MOVE group (MOVE-G) treatment versus MOVE individual (MOVE-I) treatment in four community-based outpatient treatment centres in Denmark.

**Methods:**

A two-arm non-inferiority trial comparing MOVE-I (Pedersen et al., Drug Alcohol Depend 218:108363, 2020) with MOVE-G a combined group treatment for both alcohol use disorder and drug use disorder. The primary objective is to examine whether MOVE-G is non-inferior to MOVE-I in relation to abstinence from drug and/or alcohol, number of sessions received, and completion of treatment as planned. All participants will receive treatment based on cognitive behavioral therapy and motivational interviewing, vouchers for attendance and text reminders, as well as medication as needed (MOVE). Participants (*n* = 300) will be recruited over a one-year period at four public treatment centers in four Danish municipalities. A short screening will determine eligibility and randomization status. Hereafter, participants will be randomized to the two treatment arms. A thorough baseline assessment will be conducted approximately 1 week after randomization. Follow-up assessments will be conducted at 9 months post-randomization. In addition, patients’ use of drugs and alcohol, and patients’ wellbeing will be measured in all sessions. The main outcome measures are drug and alcohol intake at 9 months follow-up, number of sessions attended, and dropout from treatment.

**Discussion:**

The present study will examine the potential and efficacy of combined groups (patients with alcohol and drug disorders in the same group) versus individually based treatment both based on the treatment method MOVE (Pedersen et al., Drug Alcohol Depend 218:108363, 2020).

**Trial registration:**

ISRCTN88025085, registration date 30/06/2020.

**Supplementary Information:**

The online version contains supplementary material available at 10.1186/s12889-021-10271-4.

## Background

Substance use disorders contribute substantially to the global burden of illness, including alcohol use disorders [1], cannabis use disorders [2], and stimulant use disorders [3]. While opioid use disorders have recently been the focus of substantial attention [4, 5], alcohol and other non-opioid drugs of abuse present their own challenges. Due to established negative consequences in a range of areas such as academic performance, physical health, mental health, family relations, social and economic consequences, and delinquency [6, 7, 8], delivering high quality treatment for substance use disorders is of crucial importance.

Individual therapy is still the most used form of treatment for substance use disorders. There is, however, increasing knowledge that supports the use of group therapy in substance use disorder treatment. Firstly, there are indications that group treatment is more cost-effective than individual treatment [6], which means that treatment providers could save time and money by implementing this treatment form. Further, a number of studies have shown that group counselling is equally or more effective compared with individual counselling. In a study examining the effects of group and individual counselling, Weiss and colleagues concluded that “…treatment outcome studies did not demonstrate differences between group and individual modalities, and no single type of group therapy reliably demonstrated greater efficacy than others.” [9]. Also, a large observational study that examined treatment results from 7800 patients [10] concluded that a larger proportion of patients in group therapy had increased likelihood for improved measures of treatment performance compared to patients in individual therapy. In a recent meta analytical review of group therapy for substance abuse disorder in adults [11], thirty-three studies with measures of group therapy outcomes were included. Significant, but small, effects of group therapy were found on abstinence compared to individual therapy, indicating that group therapy may be a little more effective than individual therapy in relation to abstinence. No significant effects were found on substance use frequency, attrition, abstinence rates, wellbeing, and substance use symptoms when compared with individual therapy, which in part was due to the small number of studies for each comparison. However, in some studies that include patients with certain co-morbid mental health disorders, such as patients with post-traumatic stress disorder, individual therapy has been found to be superior to group therapy [12].

Overall, research on outcomes in specific patient groups is still very limited and this lack of research is even more pronounced in relation to combined groups (i.e. groups that include both patients with drug use disorders and patients with alcohol use disorders), since the majority of group treatment studies only include patients with one of the disorders [11]. Specifically, there is a need for RCT studies that examine the relative usefulness of group and individual treatment formats.

### The Danish context

In Denmark, treatment for alcohol use disorders and drug use disorders is divided into two independent systems with different traditions and legislation (i.e. the health Act (alcohol treatment) and the Social Service Act (drug treatment). In the beginning of this century, efforts have been made to streamline the administrative and legislative framework for providing treatment for substance use disorders. For instance, all municipalities must provide a specific treatment offer within 14 days after treatment-seeking, treatment is free of charge, and clients can always choose a similar treatment offer from a different service provider. However, due to other differences in the legislation and administration across the drug and alcohol treatment systems, central challenges still remain. For instance, there are great differences in the legislation on assessment and the structure of provided treatment, where the legislation for drug treatment is much more detailed and comprehensive. In effect, this could mean that the quality of treatment differs. Further, In most municipalities alcohol disorder treatment and drug use disorder treatment are offered in different departments with very different treatments and treatment foci. Given the high co-occurrence of alcohol and other drug use disorders in Denmark [13, 14], service providers that specialize in just one type of use may overlook a substantial substance use problems in a number of patients, including important aspects of the condition that they are trying to treat (e.g., providing treatment for an alcohol use disorder while failing to address or assess a co-existing cannabis use disorder [15]).

Recently, in spite of different legislations, treatment of alcohol use disorder and drug use disorder treatment is increasingly carried out at the same treatment locations by the same counselors, and sometimes (although rarely) patients with alcohol use disorder and drug use disorders participate in the same counseling groups. In order to evaluate the potential of combined drug and alcohol treatment in groups, there is a strong need to improve our knowledge about the effect of combined treatment of alcohol use disorder and drug use disorder in groups compared to individual treatment.

### Treatment methods and effect in substance use treatment

A large body of research show that combinations of treatment methods may enhance treatment effect. It is well established that Motivational interviewing (MI)/Motivational Enhancement Therapy (MET) and Cognitive Behavioral Therapy (CBT) is effective in treatment of both patients with alcohol use disorder and drug use disorder [16]. MI has been combined with CBT to reduce cocaine use [17] and marijuana use [18], and results indicate that CBT treatments that incorporate MI show superior results compared to control conditions. In addition, the specific combination of both MI and CBT has been shown to be efficacious for specific sub-populations, such as people with comorbid alcohol use disorder and depression [19] and veterans with substance use disorders and problems controlling aggressive behavior [13].

However, despite the use of evidence-based treatment modalities, dropout from treatment remains a significant challenge [14]. Over the last decades, Contingency Management (CM) has gained support for improving retention and treatment for a wide range of substance use disorders, and specifically, voucher-based reinforcement therapy (VBRT) for desirable behaviors is increasingly being tested in community treatment settings as an add-on to treatment with promising results [15]. The combination of MI, CBT, and CM has proven to be one of the most promising treatment methods across ages [20]. In the summary of their Cochrane Review, Gates and colleagues conclude that “…available evidence shows the most consistent support for a combination of MET and CBT-based cannabis interventions with the adjunct of CM-abs when possible” [21].

## Objectives

The primary aim of the COMDAT trial is to test the efficacy, feasibility and acceptability of MOVE-G; a treatment that combines MI, CBT, and CM in a group format offered to patients with either an alcohol use disorder, a drug use disorder, or both. The COMDAT trial will test whether MOVE-G is non-inferior to MOVE-I in relation to abstinence from drug and/or alcohol use, number of sessions received, and completion of treatment as planned.

## Trial design

**Fig. 1:**
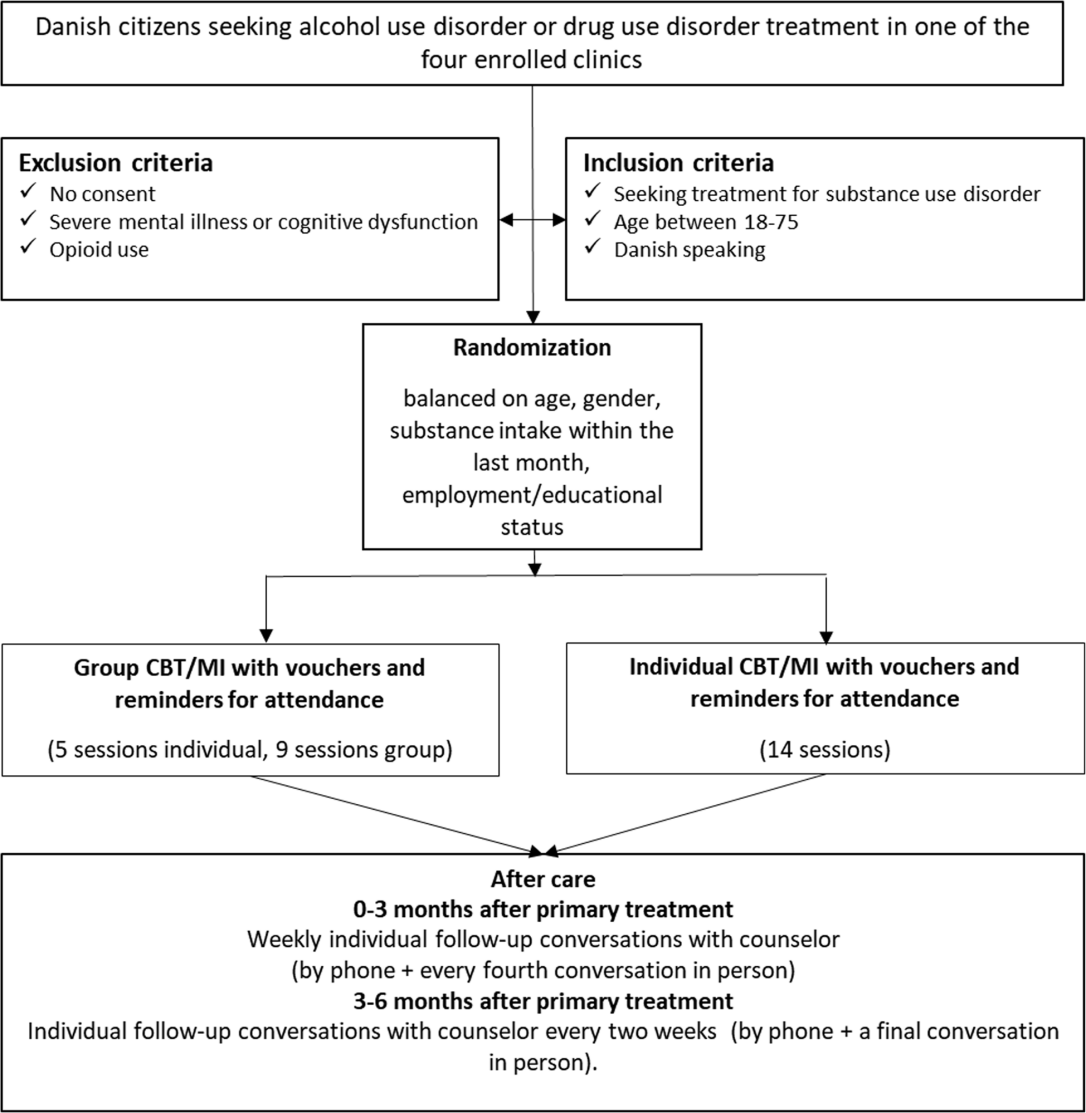
Study design

The trial design involves two arms (see Fig. 1).

## Methods: participants, interventions and outcomes

### Study setting

Participants are recruited from four outpatient municipal substance abuse treatment centers situated in four Danish cities: Herning, Randers, Aarhus, and Aabenraa.

### Eligibility criteria

Each individual seeking treatment in any of the four participating clinics (not transferring from prison or another drug treatment service) will be screened by the assessment nurse/social worker for study eligibility. It is important to note that only a selection of counsellors in the four treatment centers will be trained in MOVE-I and MOVE-G. Therefore, it is likely that some individuals who are eligible for study participation will not be asked to participate in the study due to lack of staff resources, since by Danish law individuals are not allowed to wait more than 14 days after contacting the service before being offered treatment.

### Inclusion criteria

Participants aged ≥18 years, seeking treatment for a substance use disorder, and willing to give informed consent. Participants must be able to speak and read Danish.

### Exclusion criteria

Individuals with an ongoing or recent (< 1 month) treatment episode for substance use disorders, individuals who reported using opioids for more than 9 days in the past 30 days, individuals with severe psychiatric illness, such as acute delusional, paranoid and/or psychotic disorder, acute bipolar disorder, or severe cognitive impairment. Individuals who wish to be anonymous, and who do not wish to give up any personal information to the research team, so that we will not be able to obtain any information on them subsequently.

## Interventions

The Counselors who will provide MOVE I and G at the four sites are selected by the facility managers, based on their ability and resources (time, normal work routine) to participate in the project. This approach is pragmatic and mirrors clinical practice in substance treatment services across Denmark, and would thus be possible to replicate in a potential future national implementation. During the trial, the counsellors will frequently discuss the implementation of the two conditions, and will be supervised by a clinical psychologist with expertise in CBT and MI.

**Fig. 2:**
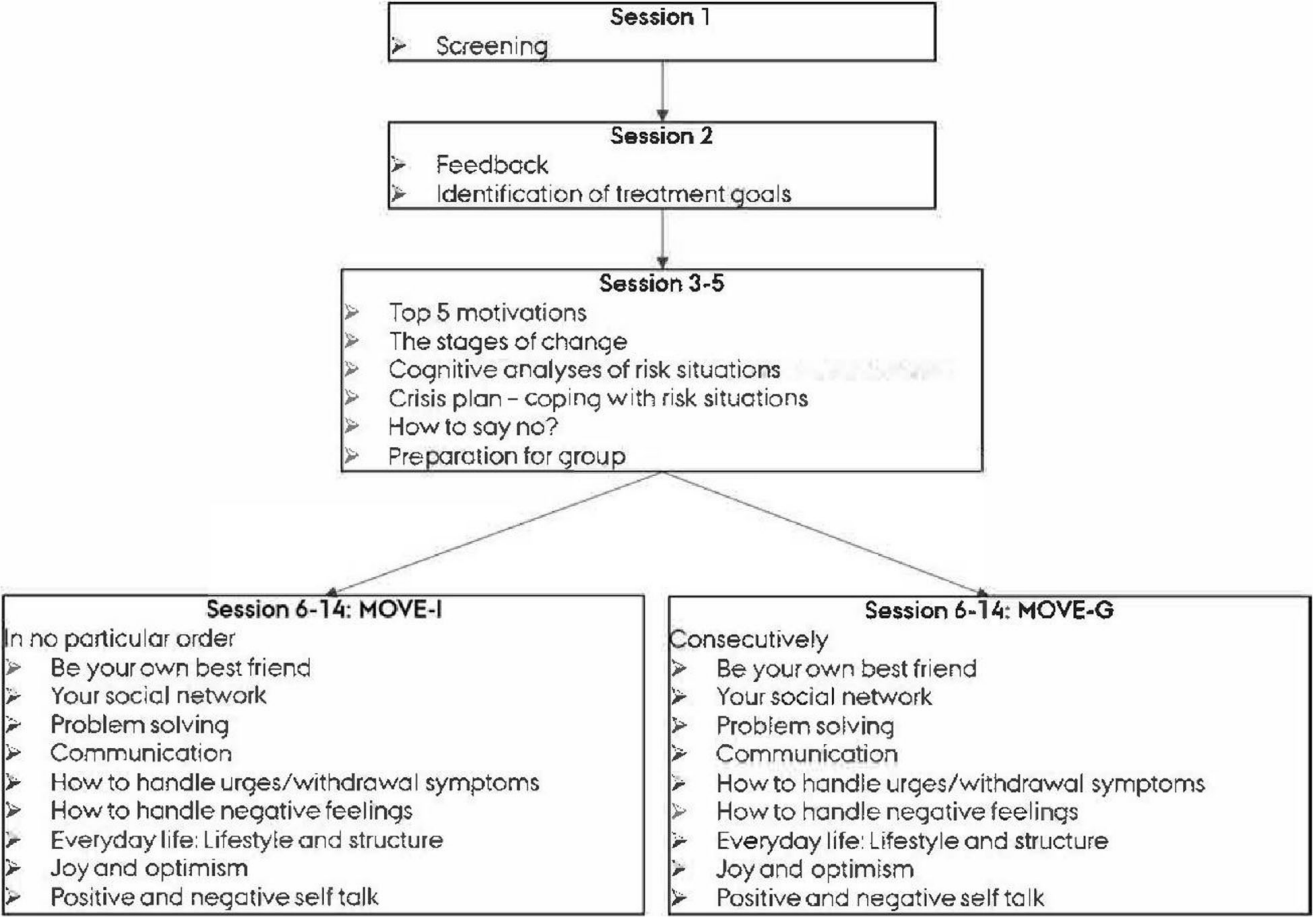
Treatment content

The two treatment conditions will include the following components: Baseline assessment and treatment planning in accordance with the Danish legislation (session 1 and 2, see Fig. 2), routine outcome monitoring, text reminders to attend all sessions, and vouchers worth 27 Euros handed out at every second session. Further, the two conditions both include three initial individual sessions of integrated MI and CBT (sessions three to five), where the primary focus will be how to handle high-risk situations and potential slips and relapses.

In the following nine sessions, both conditions will include themes that are relevant for substance use disorders. The content of the sessions is the same across the two conditions, but will be structured differently, in order to adapt to an individual and group format respectively. In MOVE-I, the order and content of the nine sessions will depend on individual treatment goals and needs. For instance, if sleep and structure is particularly important for a particular participant, the counselor may choose to use the theme “Everyday life: lifestyle and structure” in two sessions, and skip another theme that is less relevant for this participant. In MOVE-G, the nine sessions will be conducted in the succession described in the group manual.

Due to potential dropout and fluctuation of study participants, the groups in MOVE-G will have an open intake. The use of open intake in groups reflects the current clinical practice in group therapy for substance use disorders in Denmark, as well as and internationally [22, 23]. Using an open intake means that not all participants will begin with the same session, and receiving information and exercises in different order may present a potential limitation. However, we believe that the focus on handling risk situations and preventing relapse in the initial individual sessions will reduce the effect of this limitation substantially.

### After care

After completing MOVE-I or MOVE-G, participants will continue in after care conducted in the same format for both conditions; weekly individual conversations with the counsellor who provided the initial five individual sessions. In the first 3 months, the sessions will be conducted by phone/video calling platform plus every fourth conversation in person. In the next 3 months, the session will be conducted by phone/video calling platform every second week. The after care treatment is terminated with an in-person conversation with the counsellor.

### Pharmacotherapy

Pharmacotherapy for substance use disorders will be delivered as treatment as usual by the physician at each treatment site, and will be based on treatment needs and patient preference. Patients who need detoxification from alcohol will complete detoxification before attending the trial sessions, whereas pharmacological treatments, including disulfiram or acamprosate, can be provided both before and during the MOVE-G or MOVE-I treatment. Treatment for mental health problems will be available to all participants via the public health insurance in Denmark, either through the general practitioner or specialist services within the health care services.

### Outcomes

The primary outcomes are:

- ➢ Substance intake measured in all attended sessions using the Wellbeing and Outcome Monitoring (WOM) screening tool [24, 25]. In addition, participants will be asked about substance use within the last month (YouthMap/AdultMap) at enrolment and 9 months after enrolment [26–28].
- ➢ Number of sessions attended measured consecutively by the counsellor responsible for the treatment offered to the participant.
- ➢ Dropout from treatment as defined by being discharged for any reason other than having completed the primary treatment course.

The secondary outcome measures are:

- ➢ Psychological wellbeing (general, social contexts, close relationships) measured at each session using three items from the WOM screening.
- ➢ Everyday functioning (concentration, planning, keeping appointments, sleep, eating habits, cleaning etc.) within the last month measured at enrolment and 9 months after enrolment (YouthMap/AdultMap).
- ➢ Illegal activities last month at enrolment and 9 months after enrolment (YouthMap/AdultMap).
- ➢ Employment / education activities within the last month and 9 months after enrolment (YouthMap/AdultMap).
- ➢ Treatment fidelity (counsellors) measured by audio recording all sessions.

### Sample size

For the COMDAT trial, power is estimated for three outcomes related to primary treatment: Proportion of participants who complete treatment, proportion of participants who do not use illegal drugs within last week before recorded session (measured by WOM), and number of sessions attended. All calculations are based on an alpha of 0.05 and a power of 0.8. Power is calculated for a non-inferiority trial, as MOVE-I has already proved to be an effective form of treatment [29, 30].

The sample size needed for assessing completion of primary treatment is calculated based on a 49% completion rate from the MOVE-I trial and a 34% completion rate based on the national register of drug and alcohol treatment (SIB and NAB respectively) for 2017 [29], selected to match the inclusion criteria of the COMDAT trial. The NIS margin was set at 20% (app. 0.1). Based on these parameters, a sample size of 61 in each group is needed to evaluate this outcome.

For drug use at last recorded WOM session, a proportion of 41% vs. 31% was chosen based on ‘worst case’ from the previous MOVE trial. The NIS margin was set at 20% or 0.08. Based on these parameters, a sample size of 87 in each group is needed to evaluate this outcome.

For number of sessions attended power was based on a poisson regression of number of sessions attended in the MOVE-G and other groups from the MOVE trial with a base outcome of 0.7 and an IRR of 1.5. Based on these parameters, a sample size of 80 is needed in each group to evaluate this outcome. All power calculations were carried out using the packages TrialSize and WebPower for R (v. 3.6.1.)

### Assignment of interventions

Randomization will be performed by means of the minimization method, using Minim randomization software, which is a biased-coin approach with a probability of 0.7 to 0.8 for allocation of the “best fitting” treatment [31]. The minimization method was chosen to obtain an overall balanced distribution of participants, as the number of expected participants was too small for true randomization (e.g. a coin toss or random numbers generator). Furthermore, participants will be randomized at each site, in order to obtain an equal distribution within each treatment center/municipality.

## Data collection, management and analysis

### Data collection

At the first in-person contact to the participating treatment sites, a nurse/social worker will assess whether the individual is eligible for study participation. If the individual is eligible, the employee will be verbally informed about the trial and will receive a participant information sheet. If the individual provides informed consent, the same employee will collect the necessary information for randomization via an online screening questionnaire, which is automatically forwarded to the Centre for Alcohol and Drug Research, Aarhus University, in an anonymized and secured email. After the randomization, the study participant will be assigned to one of the two treatment conditions and referred to a counsellor who is responsible for the condition.

At the first counselling session after study enrolment, all participants will be assessed using the YouthMap assessment form [32] or the AdultMap assessment form, depending on the age of the participants. The YouthMap and AdultMap assessment forms are comprehensive assessment tools tailored for patients in treatment for substance use disorders. The AdultMap is almost identical to the YouthMap with the exception of a few additional items in the AdultMap that are related to children, whereas the YouthMAP has a few additional items related to education that are not in the AdultMAP.

Follow-up assessments will be conducted via telephone interviews at 9 months post-randomization. In addition, participants will fill out the WOM screening tool in all attended sessions. WOM consists of eleven items concerning current substance use and wellbeing. All sessions will be audio recorded, and a selection of these recordings will be analysed by using a revised version of the MD3-SBIRT scale that incorporates elements of CBT and MI to assess fidelity and skill level in counsellors working in outpatient substance use treatment [33].

### Data management

Quantitative data from the randomization, the YouthMap/AdultMap survey, and the session registrations will be entered into a SurveyXact server hosted on a secured server in Denmark. Quantitative data from WOM will be entered into a secure separate server hosted by Aarhus University. The Centre for Alcohol and Drug Research, Aarhus University will design and set up the databases, and host and maintain them throughout the trial. At the end of the trial, all data will be uploaded to a server on Statistics Denmark to allow merging with national registers. All audio files will be transferred by trusted employees at the four participating sites to a secured server hosted by Aarhus University.

### Statistical analyses

Quantitative data analyses will be performed in STATA V16.0. All descriptive analyses, the recruitment rate, the consent rate, loss to follow-up, departures from randomized treatment, and the prevalence of serious adverse events will be reported post-randomization and summarized by treatment arm over the course of the study. All causes of withdrawal from randomized treatment will be reported. All of this information will be provided in a CONSORT flowchart.

Summaries will be presented as means and standard deviation of variables that are approximately normally distributed, or as medians and IQRs for skewed variables. Categorical variables will be summarized as frequencies and percentages. Transformations will be used when distributional assumptions are not fulfilled for inferential tests on a continuous measure. We will examine and account for the influence of clustering at the site level on the outcomes. All models will adjust for stratification factors and randomized treatment.

The primary analyses of efficacy will be based on the intention-to-treat sample, utilizing all available follow-up data from all randomized participants. All randomized participants will be analyzed within the treatment arm to which they were originally allocated after randomization, regardless of whether they completed that specific treatment course or not. Participants who withdraw their consent for use of their data during the trial period will not be included in any of the analyses.

The main objective of the statistical analyses is to assess the equivalence of individual versus group-based treatment on substance use 9 months post-randomization. A total score for this period will be calculated for each participant. This summary score will be analyzed within a generalized linear model (GLM) framework, specifying an ordinal outcome. In Stata GSEM or a mixed effects GLM will allow for the clustering effects.

For sessions attended, a GSEM will be estimated specifying a Poisson outcome. For time to offending, a Weibull regression model will be estimated using sessions attended as a mediator.

## Discussion

The aim of this trial is to contribute to the development of a more structured, transparent, professional, and effective treatment of patients with alcohol use disorder and drug use disorder. By conducting a manualized, evidence-based comparison for the MOVE-I and MOVE-G intervention, we set the standard for showing efficacy in a sample that includes patients with one or both disorders.

This trial will be the first to evaluate the efficacy of MOVE-G including patients with alcohol use disorder and drug use disorder in the same group versus MOVE-I. The MOVE program has been shown to be easy to learn and implement. MOVE-I has shown very promising results, and has already been implemented in more than 20 Danish municipal treatment centers [34]. If the effect of MOVE-G turns out to be superior or similar to MOVE-I, it will have strong implications for clinical practice in outpatient substance abuse treatment settings in Denmark. Firstly, implementation of group counseling could lead to ressource gains in the respective treatment centers. Also, some individuals prefer receiving treatment in a group format [35, 36], and preference of treatment is an important factor for treatment effect [37, 38]. It is thus possible that providing treatment that meets individual preferences would generate even better treatment results.

## Supplementary Information

**Additional file 1: Appendix 1.** Participant information (translated version). **Appendix 2.** Schedule of enrolment, interventions, and assessments.

## Data Availability

After the conclusion of the study, data from the study will be made available by the Centre for Alcohol and Drug Research, Aarhus University, upon reasonable request, and within the limitations set by the Danish data protection legislation and regulation.

## Abbreviations

GLM: Generalized Linear Model
COMDAT: Combined Drug and Alcohol Treatment
MOVE-G: MOVE group
MOVE-I: MOVE individual
MI: Motivational interviewing
ME: Motivational enhancement
CBT): Cognitive Behavoral Therapy
CM: Contingency Management
VBRT: Voucher-based reinforcement therapy
WOM: Wellbeing outcome monitoring
