# Supplementary material for: Group versus individual treatment for substance use disorders: a study protocol for the COMDAT trial
Source: BMC Public Health. 2021 Feb 26;21:413. doi: 10.1186/s12889-021-10271-4 (PMC7913269; doi:10.1186/s12889-021-10271-4)
Supplement: Supplementary file 1 — Additional file 1: Appendix 1. Participant information (translated version). Appendix 2. Schedule of enrolment, interventions, and assessments. [file 12889_2021_10271_MOESM1_ESM.docx]

**Appendix 1.**

Participant information (translated version):

Project Combined Drug and Alcohol Treatment (COMDAT) -The MOVE Individual/group trial

In this treatment center we are testing a new treatment form for individuals with alcohol or drug-related problems. This treatment form is called MOVE. The treatment components in MOVE has previously been tested in many international trials and a Danish trial. The Danish trial, which was aimed at individuals from 18 to 25 years with substance abuse, showed very good treatment effects and participants reported being very content with participating in the study.

Based on these promising results, it is very relevant to test a treatment form that is aimed at adults (18 to 70 years), who have lost the control over their use of drugs and/or alcohol.

We are therefore asking for your participation in a scientific study managed by Professor Mads Uffe Pedersen, Centre for Alcohol and Drug Research, Aarhus University.

Before you decide on your participation in this study, you need to understand the aim and the content of the study. Therefore, please take some time to read through the following information.

You need to know that if you need to talk to a family member, a friend or anyone else before you decide on your participation, this is okay. You do not need to decide today.

**Background and aim**

The aim of this study is to examine the effect of two different treatment methods; MOVE individual= MOVE (I) and MOVE group= MOVE (G), so that people who are experiencing problems related to their use of alcohol and/or drugs can be offered the best treatment possible.

The treatment will take place in four different municipality treatment centers, and 300 people who will seek treatment for alcohol/and or drug-related problems in these four centers will be offered participation in the study.

In each treatment center, participants are randomly divided into two groups. For these two groups the first five sessions will be the same: five weekly individual sessions with the same counsellor. After these five sessions, one group will receive group therapy, whereas the other group continue to receive individual therapy sessions. The groups will include individuals with drug related as well as individuals with alcohol related problems.

Maybe you are a little concerned about the concept of group therapy, however, you should know that individual treatment and group treatment has been shown to be equally effective in reducing or eliminating drug and/or alcohol use.

Since a part of the study is focused on increasing the attendance rate of participants in treatment, all participants will receive a voucher worth 200 Danish kroner after every other session.

Before each session you will receive a text message with a reminder for your appointment at the center (only if you allow this).

All counselors who are participating in the study have been trained in the methods applied in the study.

In order for the researchers to be able to examine which method is more effective, all sessions will be recorded on tape. The researchers are only interested in how your counsellor is applying the treatment methods.

Your participation is an important contribution in our work to examine the effect of alcohol and drug treatment in Denmark. Participation is voluntary. You may at any time and without any reason withdraw your consent. This will not have any consequences for your treatment and you will be transferred to standard treatment.

**Study overview**

The treatment course consists of primary treatment (14 sessions) and after treatment (6 months), where contact with the treatment center is gradually reduced. All participants will receive treatment with MOVE and will receive vouchers at every second treatment attendance (only in the primary treatment). The after treatment consists of two phone conversations + two personal conversations per month in the first three months. In the next three months the after treatment consists of one phone conversation every second week. The after treatment is concluded with an in-person conversation with the counsellor.

During the treatment course you will be interviewed one time by an employee from The Center for Drug and Alcohol research at Aarhus University. The interview will take place nine months after you have been enrolled in treatment. After the interview you will receive a voucher worth 100 Danish kroner. You can only be enrolled in the study one time.

*What kind of treatment would you get if you did not wish to participate in this study?*

If you do not want to participate you will be offered the treatment that is normally offered (standard treatment). You will not be offered fewer sessions if you do not wish to participate. You will have the same opportunities to talk to doctors, psychiatrists, social workers etc. as participants in the COMDAT trial. However, if you do not wish to participate in the trial it is not certain that you will be offered the MOVE treatment method or vouchers for attendance.

Several treatment centers use group therapy as standard treatment and it is therefore possible that you will also be offered this type of treatment if you do not wish to participate in the COMDAT trial. There is no risk associated with participation in the study since you will not be offered less or worse treatment than by not participating in the study.

**What is your gain?**

- You will be part of a treatment course that we know that others have been satisfied with, and that has shown promising results.
- You receive vouchers worth 200 Danish kroner at every other attendance.
- As part of the study you will be interviewed one time by an employee from The Center for Drug and Alcohol research at Aarhus University. The interview will take place nine months after you have been enrolled in treatment. After the interview you will receive a voucher worth 100 Danish kroner.
- If you are interested you can get information on study results
- You support the development of drug and alcohol treatment in Denmark.

**Protection of your personal information**

In the COMDAT trial we adhere to the data protection law and regulations. This means that researchers are obliged to keep all information confidential. The researchers may not pass information about individuals in the study, to people who are not a part of the small group of researchers conducting the study.

This means that all data about you and your treatment will be placed on a safe server at Aarhus University. Your data will be encrypted (will not be readable/available), and a two factor password will be used to get access. It is only researchers in the study who will get access to the data. As soon as all data have been collected they are sent to Statistics Denmark and will be anonymized so that even the researchers will not be able to identify participating individuals. In other words, after data has been anonymized no one will be able to tell who you are. You can find more about the data protection law and regulations via this link: https://www.datatilsynet.dk/generelt-om-databeskyttelse/lovgivning/

If at any time, you regret your participation in the study, you can have all the personal information that you have given deleted from the servers at Aarhus University. This also applies to taped conversations.

The COMDAT trial is funded by the TRYG foundation and is conducted by The Center for Drug and Alcohol Research, Aarhus University. The budget is 1 million Danish kroner for conducting the study through development of a treatment manual, interviewing participants, data management, data analyses and writing a research report. The researchers have no particular attachment to the financial contributor and the researchers who are managing the study are full-time employees at Aarhus University.

When data have been collected and analyzed, the results will be published. We expect to publish results during 2022. In our publications all results will be anonymous and your information will not be recognizable.

We hope that you feel that you have sufficient knowledge on what participation in the COMDAT trail means, and that you feel able to decide whether you wish to participate in this trail or not.

We also ask you to read the attached material: ”study participants´ rights in a health scientific research project”.

If you want to know more about the study, you are welcome to contact Mads Uffe Pedersen, Center for drug and alchol research, Aarhus Universitet, Bartholins Allé 10, bygning 1322,2., 8000 Århus C. mup@crf.au.dk, +45 8716 5771.

**Resume**

All participants in the study is randomly divided in two groups.

All participants receive one weekly individual session with a counsellor the first five weeks of the treatment course. After this, one group continues to group treatment (9 sessions) and the other group continues to receive individual treatment (9 sessions).

For all participants, the treatment course ends with an after treatment course, where the contact to the treatment center is gradually reduced.

All participants receive vouchers worth 200 Danish kroner at every other session attendance (only primary treatment: 14 sessions)

Text messages will remind you of appointments

All sessions will be taped in order for researchers to examine if the counsellors are applying the methods correctly.

All participants will be interviewed one time by an employee from The Center for Drug and Alcohol Research at Aarhus University and will receive a voucher worth 100 Danish kroner after the interview.

If you decide to participate in the study we would like you to sign a consent form. Remember that you have the right to take some time to consider before you sign the consent form.

Best regards,

**Mads Uffe Pedersen** **Sidsel Karsberg**

Professor Assistant Professor

| *Aarhus University*  *Business and Social Sciences*  *Centre for Alcohol and Drug Research* |
| --- |
|  |

**Participant Declaration:**

I have received oral and written information, and I have sufficient knowledge about purpose, methods, gains and disadvantages to sign the participant consent form.

I know that it is voluntary to participate in the study and that I can always withdraw my consent without losing my current or future rights for treatment.

I give consent to participate in the COMDAT research project and I have received a copy of this participant information sheet for my own use.

Name:

Date:

Signature:

Do you wish to be informed about the results of the study (tick)?

Yes:

No:

**Appendix 2. Schedule of enrolment, interventions, and assessments**

|  | **STUDY PERIOD** | | | | | |
| --- | --- | --- | --- | --- | --- | --- |
|  | **Enrolment** | **Allocation** | **Primary intervention** | | | **After care** |
| **TIMEPOINT**** | ***-t_1_*** | **0** | ***Session 1*** | ***Sessions 2-5*** | ***Sessions 6-14*** | ***Sessions 15-32*** |
| **ENROLMENT:** |  |  |  |  |  |  |
| **Eligibility screen** | X |  |  |  |  |  |
| **Informed consent** | X |  |  |  |  |  |
| ***No other procedures*** | X |  |  |  |  |  |
| **Allocation** |  | X |  |  |  |  |
| **INTERVENTIONS:** |  |  |  |  |  |  |
| ***MOVE-I*** |  |  | YouthMAP screening | Treatment planning and work on high-risk situations for drug use | Working with relevant themes and individual goals | Working with individual goals and risk situations |
| ***MOVE-G*** |  |  | YouthMAP screening | Treatment planning and work on high-risk situations for drug use | Working with relevant themes in group | Working with individual goals and risk situations |
| **ASSESSMENTS:** |  |  | YouthMap+WOM^^[[1]](#footnote-1)^^ | WOM | WOM | WOM + YouthMAP  (9 Months) |
| ***Outcome variables*** |  |  | Current well-being and substance use | Current well-being and substance use | Current well-being and substance use | Retention in treatment |
| ***Other variables*** |  |  | Attendance | Attendance | Attendance | Attendance |

1. The WOM screening is used in all attended sessions [↑](#footnote-ref-1)
